# Combination of Neoadjuvant Therapy and Liver Transplantation in Pediatric Multisystem Langerhans Cell Histiocytosis With Liver Involvement

**DOI:** 10.3389/fonc.2020.566987

**Published:** 2020-10-07

**Authors:** Chen Chen, Guangxiang Gu, Tao Zhou, Mingzhu Huang, Qiang Xia

**Affiliations:** Department of Liver Surgery and Liver Transplantation Center, Renji Hospital, Affiliated to Shanghai Jiao Tong University School of Medicine, Shanghai, China

**Keywords:** Langerhans cell histiocytosis (LCH), neoadjuvant therapy, liver transplantation, follow-up, multisystem

## Abstract

**Background:** Langerhans cell histiocytosis (LCH) is characterized by misguided myeloid differentiation, whose prognosis was poor with involvement of risk organs. Remaining issues include how to improve the outcomes of patients with risk-organ involvement.

**Methods:** A retrospective study was conducted in Renji Hospital exploring the effects of neoadjuvant therapy in combination with pediatric liver transplantation (LT) for LCH patients based on data collected between October 2006 and October 2019.

**Results:** We presented here five cases of multisystem LCH patients underwent systemic chemotherapy to control active lesions, followed by LT to treat end-stage liver diseases. Manifestations before LT included elevated transaminase levels (*n* = 5, 100%), jaundice (*n* = 4, 80%), ascites (*n* = 3, 60%), and variceal hemorrhage (*n* = 1, 20%). Three patients underwent orthotopic liver transplantation (OLT) and two underwent living donor liver transplantation (LDLT). Until December 2019, median follow-up time was 32 months (range, 2–67 months). Liver functions significantly improved compared with pre-operative conditions. One patient had perioperative hepatic artery complications and one patient had a recurrence in the lung. EBV infection occurred in four (80%) patients and CMV infection occurred in one (20%). There was one case of drug-induced liver injury diagnosed on biopsy 13 months after LT. None underwent re-transplantation and there were no rejection or portal vein and biliary complications.

**Conclusion:** Combination of neoadjuvant therapy and LT is an effective paradigm in treatment of multisystem LCH with severe liver dysfunction. With advances in chemotherapy regimen for multisystem LCH and LT surgery, perspectives on prognosis for LCH children are promising.

## Introduction

Langerhans cell histiocytosis (LCH) is a rare neoplasm of myeloid precursor cells, derived from cells from monocyte, macrophage, and dendritic cell lineages (1–3). The annual incidence of LCH is ~1 per 200,000 children (4). For patients with liver involvement, the risk of death is three times greater than those without (5).

Infiltrating the skin, liver, spleen, bone marrow, lung, or other systems, there have been more than 100 subtypes described since the first classification in 1987, with a wide range of presentations (6). Therapeutic strategies to LCH patients were often tailored to individuals according to stratification of high- or low- risk groups (7, 8). Liver, spleen, and bone marrow are thought to be the organs most at risk. Multisystem LCH patients show poor prognosis, especially congenital cases (9). Although systemic chemotherapy, the mortality rate of patients exhibiting risk organs remain high at 16–38% (10).

For LCH patients with liver involvement, portal infiltrations can lead to periportal fibrosis and bile duct destruction, resulting portal hypertension, and cirrhosis (10). However, it is clear that children with multiorgan diseases are contraindicated for LT (11). Treatment for these multisystem LCH patients with end-stage liver problems should not be restricted to single method. Limited data on neoadjuvant techniques in combination with pediatric LT have been published since 1990s. Recent years, there still has been no satisfactory outcomes for multisystem childhood LCH patient with liver involvement. Therefore, to determine the effect of combination of neoadjuvant therapy and LT for pediatric multisystem LCH patients, medical records of children with a diagnosis of LCH in our transplant center were retrospectively reviewed.

## Patients and Methods

The retrospective single-center study included all LCH patients who have undergone LT at Renji Hospital, Shanghai Jiao Tong University School of Medicine from October 2006 to October 2019. All operations were approved by review board. Informed consent concerning the permission to collect and use data was obtained from all children's guardians.

Organ involvement was defined as follows: for the liver, alanine transaminase (ALT), aspartate transaminase (AST), and bilirubin increase or albumin decrease or liver biopsy confirmed LCH histology characteristics; for the spleen, splenomegaly indicated by ultrasonography (≥2 cm under the costal margin); for the hematopoietic system, the presence of thrombocytopenia or leukopenia in the peripheral blood; for the bone and lungs, findings on computed tomography (CT) scan or biopsy; for the skin, seborrheic dermatitis, or eczematous eruption confirmed by pathology; for the ear, clinical manifestations of external acoustic meatus, or purulent secretion with or without pathology evidence.

The type of transplantation included orthotopic liver transplantation (OLT) and living donor liver transplantation (LDLT). All organ donation and matching were achieved through the China Organ Transplant Response System (COTRS). Living donor evaluation was performed before operation. The determination of segments was based on 3D virtual surgery planning system (IQQA-Liver) and intraoperative findings. The left lobe with middle hepatic vein and the left lateral graft were finally chosen in two patients underwent LDLT, respectively. University of Wisconsin solution was used for organ preservation.

In our transplant center, during LT, take down ligaments around liver first, then dissect the hepatic hilum and divide hepatic arteries, common bile duct. Clamp and divide the portal vein and hepatic veins, then remove the native liver. Surgical procedures of OLT differentiate from LDLT in the way of clamping and anastomosis, with reconstruction of both suprahepatic and infrahepatic inferior vena cava in OLT. While in LDLT, dissect all short hepatic veins into the vena cava and reconstruct suprahepatic vena cava. Reconstruct hepatic vein, portal vein, hepatic artery in turn. Finally, end-to-end anastomosis or choledochojejunostomy was used to reconstruct bile duct.

The principles of follow-up are once a week in the first 3 months after discharge, once per 2weeks from the fourth to sixth month, once per month at 6 months post-operation and visits to the clinic at any time if anything abnormal should occur.

Non-normally distributed qualitative variables were described by medians with interquartile range. Differences between groups were calculated using the Student *t*-test. All statistical tests were two-sides, and *P* < 0.05 was considered significant. Analyses were undertaken using GraphPad Prism 7 (GraphPad, Version 7.0a, California).

## Results

### Neoadjuvant Therapy and Characteristics Before LT

The median age of LCH patients at diagnosis was 15 months (range, 13–28 months) (Table 1). The first symptoms lead to diagnosis of LCH were purulent secretion in ear (*n* = 2), skin rash (*n* = 2), and elevated liver enzymes discovered by accident (*n* = 1). All five patients with multisystem LCH had at least three organs involved, belonging to high-risk patients with involvement of two or more organ systems or one or more high-risk organ systems. Organ involvement included liver (*n* = 5, 100%), spleen (*n* = 5, 100%), bone (*n* = 3, 60%), skin (*n* = 3, 60%), blood system (*n* = 2, 40%), ear (*n* = 2, 40%), lung (*n* = 1, 20%) (Table 2). Patients received systemic neoadjuvant chemotherapy in different children's hospitals before LT, which consisted of an initial phase and maintenance phase (Table 1). One patient turned to second-line therapy because of poor response to initial regimen at the 6 weeks post-treatment evaluation. After evaluated as non-active in other systems, these five LCH patients underwent LT at Renji Hospital, accounting for 0.25% among 2,000 cases of pediatric LT form October 2006 to October 2019. The median age at LT was 53 months (range, 24–81 months) (Table 1). Clinical and laboratory manifestations before LT included elevated transaminase levels (*n* = 5, 100%), jaundice (*n* = 4, 80%), ascites (*n* = 3, 60%), and variceal hemorrhage (*n* = 1, 20%).

**Table 1:** Clinical characteristics and neoadjuvant therapy of children with LCH.

**Patient number**	**Age at diagnosis of LCH (years)**	**Chemotherapy before LT**	**Indications for LT**	**Age at LT (years)**	**Type of LT**
1	1.25	Initial phase: Pred (4 W, taper 2 W) +VCR (D1 of W1–6) + Ara-C (D1–4 W 1, 3, 5) Maintenance phase: Perd (D1–5 Q3W) + VCR (D1 Q3W) +6-MP (QN)	Portal hypertension (varices, splenomegaly), liver dysfunction, hypersplenism	6.8	OLT
2	1.75	Initial phase: Pred (4W, taper 2W) +VP16 (D1–5, 18, 25, 32, 39) +VBL (D15, 22, 29, 36) Maintenance phase: 6-MP (W6–52) + VBL (D1, W9–42/Q3W) + VP16 (D5, W9–42/Q3W) + MTX (D1, W9–42/Q3W) + THF (D1, W9–42/Q3W)	Portal hypertension (varices, splenomegaly), liver dysfunction	4.4	OLT
3	1.25	First-line therapy: VCR (D1 of W1–5) +VP16 (D1–3 of W3, D1 of W4–6) +VDS (QW) Second-line therapy: Ara-C+ Cladribine^a^	Portal hypertension (varices, splenomegaly)	4.3	LDLT (left lobe +MHV)
4	2.33	Initial phase: Pred + VCR + Ara-C Maintenance phase: VCR+ Pred +Thymosin Withdrawal in May, 2015	Portal hypertension (variceal hemorrhage)	6.3	OLT
5	1.08	Initial phase: VDS (W1–11) +VP16 (W1–4, W9–11) +Pred (W1–11) +Ara-c(W5–8) +CTX (W5–8) Maintenance phase: VDS (Q3W before LT)	Liver dysfunction, hypersplenism	2	LDLT (left lateral lobe)

a *First-line therapy was not effective for patient 3, who then received second-line therapy. LCH, Langerhans cell histiocytosis; LT, liver transplantation; Pred, prednisolne; VCR, vincristine; VBL, vinblastine; VP16, etoposide; VDS, vindesine; CTX, cyclophosphamide; Ara-C, cytarabine; W, week; D, day; QW, quaque week, each week; Q3W, quaque 3 week, every 3 weeks; QN, quaque nocte, each night; OLT, orthotopic liver transplantation; LDLT, living donor liver transplantation; MHV, middle hepatic vein; TAC, tacrolimus; MMF, mycophenolate mofetil*.

**Table 2:** Organs and systems involvements of LCH patients: manifestations and pre-operation imaging examinations.

**Patient number**	**Involved organs/ systems**	**Liver**	**Spleen**	**Blood system**	**Bone**	**Skin**	**Others**
1	Liver, spleen, blood system, lung, neoplasm of external acoustic meatus	Liver dysfunction: ALT 104.9 U/L, AST 172.8 U/L, TB 78.5 umol/L	Splenomegaly	WBC 3.6 × 10^∧^9/L, PLT 62 × 10^∧^9/L	None	Negative by immunohistochemistry	Lymph node biopsy through thoracoscopy and neoplasm of external acoustic canal showed features consistent with LCH
2	Liver, spleen, bone	Liver dysfunction: ALT 79 U/L, AST 178 U/L, TB 457 umol/L	Splenomegaly	None	Left temporal skull lesions immunohistochemistry indicated Langerin (+++), S-100(+++), CD1a (+++), Kp-1(–), Ki67:20% (proliferation index)	None	None
3	Liver, spleen, skin, bone	Liver dysfunction: ALT 38 U/L, AST 110 U/L, TB 43 umol/L	Splenomegaly	None	Right distal femur, left ilium, left occipital bone, frontal bone small low-density lesions	Skin of back immunohistochemistry: CD1a+, S100+, Langerin+, PGM1-, MAC387 partial +, CK-, LCA+, MPO–	None
4	Liver, spleen, skin, bone	Liver dysfunction: ALT 31 U/L, AST 131 U/L, TB 12.8 umol/L	Splenomegaly	None	Right parietal bone, frontal bone, left temporal bone and occipital bone multiple bone defects	Skin of back confirmed LCH	None
5	Liver, spleen, blood system, Skin, Ear	Liver dysfunction: ALT 159 U/L, AST 198 U/L, TB 172.2 umol/L	Splenomegaly	WBC 2.06 × 10^∧^9/L	None	Immunohistochemistry: S100–, CD163+, CD1a+, Ki67 about 30%, INI1+, CK–, EMA–, LCA+	Ear pus, but no neoplasm and no pathological evidence

There were three pediatric donors, donation after circulatory death (DCD), and two living donors. The median age of pediatric organ donors was 4 years (range, 2–6 years) and all of them were male. Both living donors were mothers, aged 36, 27 years, respectively. The blood type combinations were identical in four cases and compatible in one case.

### Operative Information

**Figure 1:**
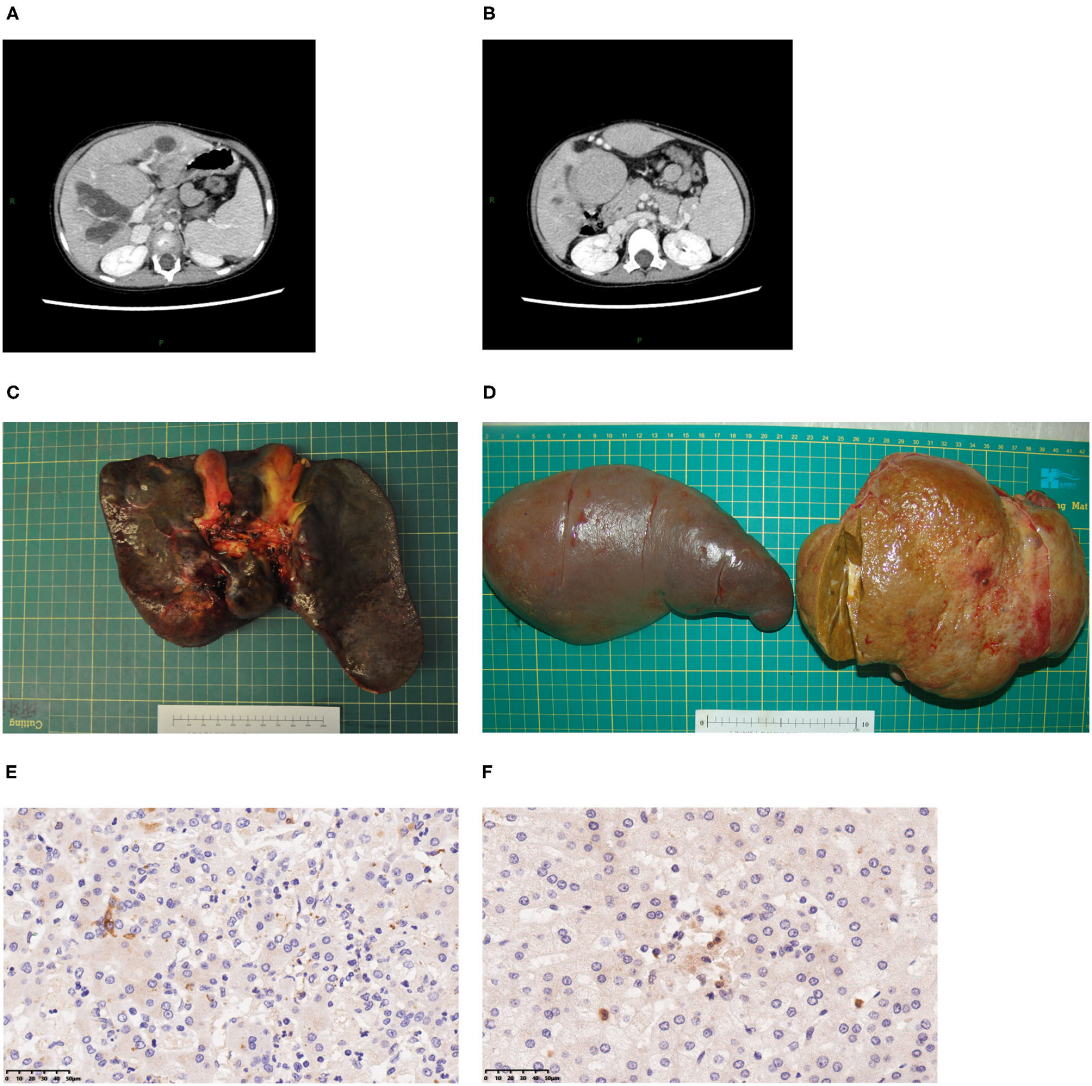
Typical clinical presentations of LT children diagnosed with LCH. **(A)** CT scan revealing dilatation of intrahepatic bile duct, splenomegaly, hepatic mass lesions thought to be dysplastic nodules. **(B)** Varicose veins. **(C)** A 2-year-old patient's liver. **(D)** A 7-year-old patient's spleen and liver. **(E)** CD1a staining of liver, 40×. **(F)** Langerin staining of LCH patient's liver, 40×.

Three patients underwent whole-liver orthotopic liver transplantation (OLT) using no organs from executed prisoners. Two underwent living donor liver transplantation (LDLT). Of these two grafts, one was a left lateral lobe, one was a left lobe with the middle hepatic vein (MHV). Patients 1 and 5 also underwent splenectomy because of hypersplenism (Figures 1C,D). Graft volume/recipient body weight ratios (GRWR) of the five patients were as follows: 1.43, 3.04, 1.86, 3.47, 2.84%. Preoperative computed tomography (CT) scan suggested dilation of intrahepatic bile ducts, space-occupying hepatic lesions, considered hyperplastic nodules or others, splenomegaly, and varicosity (Figures 1A,B), which were confirmed by operation findings. Gross examination revealed greenish-tan liver surface and some appeared micronodular on the external and cut surfaces. Histopathology of the resected liver indicated micronodular cirrhosis with interstitial fibrous proliferation and inflammatory cell infiltration. Immunohistochemistry (IHC) of paraffin-embedded liver samples showed individual CD1a-positive and Langerin-positive cells (Figures 1E,F). No IHC evidence indicated that the gallbladders had Langerhans cell infiltration.

### Post-operative Follow-Up

**Figure 2:**
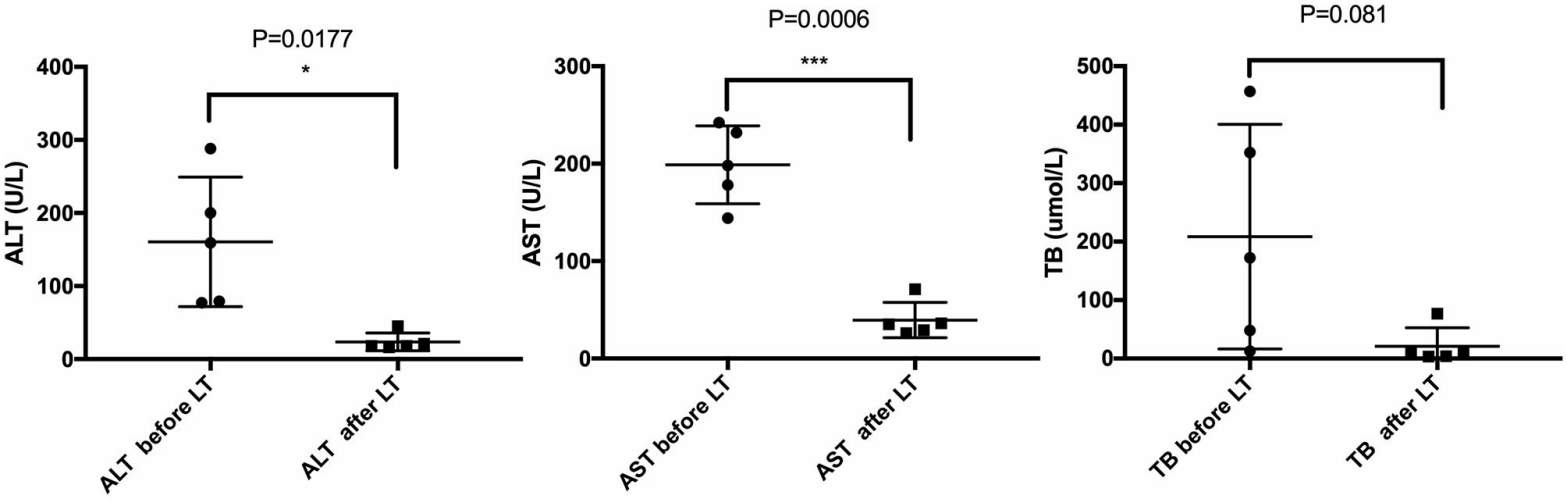
Comparison of liver function before and after LT. Most recent follow-up data with the pre-operative liver function of LCH patients. *p < 0.05; ***p < 0.001. ALT, alanine transaminase; AST, aspartate transaminase; TB, total bilirubin.

**Figure 3:**
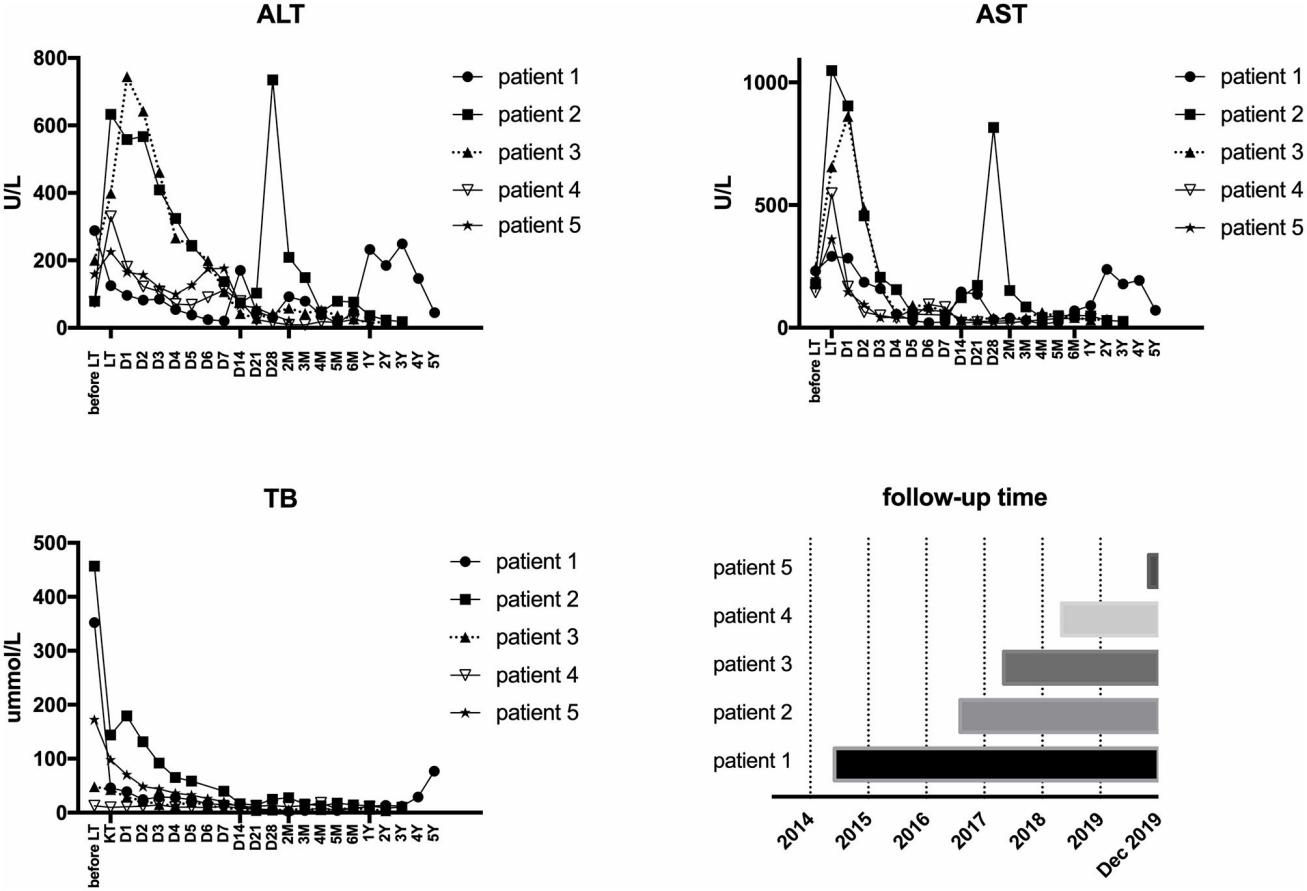
Post-operative follow-up.

All five LCH patients have been followed up as of the date of this manuscript, and none underwent re-transplantation. The median follow-up time until December 2019 was 32 months (range 2–67 months). Liver functions returned to normal within 3–4 weeks after LT. According to the most recent follow-up data, liver functions improved significantly compared with preoperative status, reflecting the significant decreases in transaminase levels (Figure 2). The type of operation did not appear to affect long-term prognosis. Immunosuppression was based on tacrolimus (TAC), together with prednisone tapers in the early period post-operation. Three patients continue to take mycophenolate mofetil in combination with TAC and one patient also took rapamycin as a supplement. No patients were administered postoperative chemotherapy. Liver function tended to remain stable over the long term (Figure 3). The surgeries caused transient elevations of transaminase levels compared to pre-operation data, both of which would decrease later. Transaminase levels of patient 2 increased 3 weeks after LT and biopsy indicated moderate liver damage with mild central perivenulitis but no rejection. After reducing the dosage of TAC from 1 mg/1.5 mg to finally 0.75 mg/0.5 mg, ALT and AST levels decreased substantially.

### Complications After LT

The first LCH patient had hepatic artery thrombosis in the early postoperative period. He had signs of soft tissue in the right lung found on PET-CT 7 months after LT that were similar to findings 4 months before LT. No obvious changes of opacities in the lung were found on CT the following year. No others had evidence of recurrence or progressive lesions. This patient had drug-induced liver injury 13 months after LT. For all five patients, no portal vein complications and biliary complications occurred. None of patients developed post-transplant lymphoproliferative disorder; however, four children had Epstein-Barr virus (EBV) infection after LT, with EBV-DNA load in venous blood exceeding 5 × 10^3^ copies/ml. One patient had cytomegalovirus (CMV) infection, with CMV-DNA load in venous blood more than 500 copies/ml. None were infected with hepatitis B virus. None had pathological diagnoses of acute rejection (Table 3). Two patients who underwent splenectomy had increased platelet counts postoperatively. There was no evidence of involvement of the urinary system, nervous system, skeleton, or mouth. No growth and development problems were found.

**Table 3:** Outcomes and complications of LCH patients after LT.

**Patient number**	**Status**	**Immunosuppressive drugs after LT**	**Rejection**	**Recurrence**	**Drug-induced injury**	**Vascular complications**	**Infections**	**Follow-up (months)**
1	Alive	TAC, MMF, rapamycin	No	Lung	Yes	Hepatic artery thrombosis	EBV	67
2	Alive	TAC, MMF	No	No	No	None	No	38
3	Alive	TAC	No	No	No	None	EBV	29
4	Alive	TAC, MMF	No	No	No	None	EBV, CMV	20
5	Alive	TAC	No	No	No	None	EBV	2

## Discussion

In this study, all five patients were multisystem childhood LCH, underwent chemotherapy prior to LT to improve preoperative status, including initial phase and maintenance phase. They all benefited from this paradigm with significant improvements of liver functions after LT. There were three underwent OLT and two underwent LDLT with left lateral lobe or left lobe with MHV graft. There were no serious vascular complications or recurrence of liver involvement. Four patients once had EBV infection and one patient had CMV infection. 2–67 months follow-up demonstrates that liver function has been stable without acute and chronic rejection.

In our study, two LCH patients had sclerosing cholangitis before therapy, manifested as repeated attacks of fever and uneven beaded changes in left and right hepatic duct as well as common bile duct by radiology. Two patients underwent LT due to irreversible abnormalities of liver function, whose total bilirubin were 352.4 and 457 umol/L before LT. One patient had decompensated cirrhosis, manifested as variceal hemorrhage.

Our study has long follow-up pediatric patients with satisfactory outcomes and demonstrate that combination of neoadjuvant therapy and liver transplantation can cure liver failure episodes and relieve LCH by experienced surgeons. Hatemi et al. summarized isolated 13 case reports of LCH with sclerosing cholangitis in adults (12). LT for end-stage liver disease with LCH children has been reported for several cases (Table 4). Our outcomes were obviously better owing to surgical experience and skills in our center. In our series, three patients who underwent OLT and two who underwent LDLT all experienced remission postoperatively. This study pointed out the safety and efficiency of LT combined neoadjuvant chemotherapy in pediatric patients with multisystem LCH and liver involvement.

**Table 4:** World report of pediatric LT for LCH patients.

**First author**	**Year**	**Country**	**No. of patients**	**Mean age at LT**	**Operation type**	**Status**	**Rejection**	**Recurrence**	**Complication**	**Follow-up**
Stieber (13)	1990	USA	2 (exclude 1 adult)	/	OLT	Alive (100%)	Yes (100%)	No	Uncontrollable rejection and re-transplantations	57–60 months
Rand and Whitington (14)	1992	USA	2	4.25 y	OLT (reduced-size)	Alive (100%)	Yes (100%)	No	Re-transplantation; CMV infection and hepatitis; gastrointestinal bleeding	30–34 months
Zandi et al. (15)	1995	France	5	13 y	OLT (2 whole graft, 3 left liver)	Alive (60%)	Yes (80%)	No	CMV infection; renal failure; ulcer bleeding; multiorgan failure	9 days−88 months
Melendez et al. (16)	1996	UK	1	2.5 y	OLT (right lobe)	Alive	Yes	No	EBV-driven lymphoproliferative disease	19 months
Newell et al. (17)	1997	USA	6	3.1 y	/	Alive (67%)	Yes (100%)	Yes (33%)	Re-transplantation (66%); PTLD	2.1–7.5 years
Hadzic et al. (18)	2000	UK	2	24 months	OLT (reduced-size)	Alive (100%)	No	Yes (100%)	PTLD	5–60 months
Braier et al. (19)	2002	Argentina	5	/	/	Alive (60%)	Yes (20%)	No	Renal failure; sepsis; bowel volvulus	14–37 months
Rajwal et al. (20)	2003	UK	1	28 months	SLT (left lateral lobe)	Alive	Yes	Yes	PTLD	16 months
Honda et al. (21)	2005	Japan	1	9 months	LDLT	Died	Yes	No	EBV infection; CMV infection; liver failure	22 months
Yuksekkaya et al. (22)	2011	Turkey	1	28 months	LDLT	Died	Yes	No	GVHD; liver failure	6 months

*SLT, split liver transplantation; OLT, orthotopic liver transplantation; PTLD, post-transplant lymphoproliferative disease; LDLT, living donor liver transplantation; GVHD, graft-versus-host disease*.

No standard therapeutic plan has been decided for LCH patients. Strategies include front line treatment and evaluation, maintenance therapy, salvage therapy (23). In the past, the most common therapy is vinblastine and corticosteroid for 6–12 weeks, tested by LCH-I, -II, and -III clinical trials (24–26). For patients exhibit involvement of risk organs, regimen of Ara-C, vincristine, and prednisolone is recommended by Japan LCH Study Group-96 (27). Targeted drugs such as vemurafenib has shown efficacy in LCH patients with BRAF-V600E mutation (28). In our study, four patients had good response status to initial phase and one case changed to second-line regimen. All of them had conditions for next step surgery after chemotherapy. On the other hand, liver transplantation cannot be performed unless chemotherapies control active lesions.

Among complications have been reported, post-LT infections and were more common than recurrence. In our study, four of the five became infected with EBV and one had CMV infection. One patient was diagnosed with recurrence of pulmonary Langerhans cell histiocytosis (PLCH). This recurrence was discovered on CT scan with typical signs of cystic lesions, that had also been present prior to LT. LCH may have an etiologic association with virus infection (22). The present study demonstrated that EBV DNA in LCH patients tissue samples was significantly greater than in control groups. Nineteen (63.33%) of 30 LCH patients had EBV DNA positive results (29). What's more, considering the admission of immunosuppressants, it is more likely for them infected with microorganisms. This suggests that the infection rate in our cohort is comparable to other studies. Nevertheless, we should remain vigilant for the development of EBV-associated diseases such as lymphoma (29). Regarding recurrence, after complete LCH resolution, the reactivation rate was 46% in 5 years in a retrospective analysis of 335 patients with multisystem LCH; (30) however, there were relatively fewer cases of recurrence in the liver. Hadzic et al. reported two patients with LCH who recurred in the grafts at 60 and 5 months after OLT (18). Although we believe that the possibility of liver recurrence is relatively low after remission, to improve outcomes in patients with multisystem disease, chemotherapy needs to be adjusted or prolonged (26).

Our study has some limitations. The number of enrolled patients is relatively small. Considering LCH is a rare disease, aggregating data is still needed to achieve a sufficient sample size to produce more reliable estimates, together with full documentation and multicenter cooperation.

In conclusion, according to our data, liver transplantation with neoadjuvant therapy is promising for LCH patients with end-stage liver disease. With advances in surgical techniques and pre-transplant management, we are more confident that LT will improve the outcomes for LCH children with liver involvement.

## Data Availability Statement

The raw data supporting the conclusions of this article will be made available by the authors, without undue reservation.

## Ethics Statement

The studies involving human participants were reviewed and approved by The Ethics Committee of Renji Hospital, School of Medicine, Shanghai Jiaotong University. Written informed consent to participate in this study was provided by the participants' legal guardian/next of kin. Written informed consent was obtained from the minor(s)' legal guardian/next of kin for the publication of any potentially identifiable images or data included in this article.

## Author Contributions

CC participated in data curation and analysis, as well as drafting the initial manuscript. GG and QX participated in research design and review or editing of the manuscript. TZ and MH provided resources of doing the investigation. All authors contributed to the article and approved the submitted version.

## Conflict of Interest

The authors declare that the research was conducted in the absence of any commercial or financial relationships that could be construed as a potential conflict of interest.
